# Supplementary material for: Epigenetic Silencing of LMX1A Contributes to Cancer Progression in Lung Cancer Cells
Source: Int J Mol Sci. 2020 Jul 30;21(15):5425. doi: 10.3390/ijms21155425 (PMC7432919; doi:10.3390/ijms21155425)
Supplement: Supplementary file 1 [file ijms-21-05425-s001.zip › ijms-852311 Supplementary Materials.pdf]

International Journal of Molecular Sciences

MDPI

Supplementary Materials

Table 2. Primer sequences used for MSP, Q-MSP, bisulfite sequencing, and qRT-PCR.

Gene	Primer Sequences	Application
LMX1A-M-R	5′-TTTAGAAGCGGGCGGGAC-3′	MSP
LMX1A-M-R	5′-CCGAATCCAAACACGCG-3′	MSP
LMX1A-U-F	5′-GAGTTTAGAAGTGGGTGGGATG-3′	MSP
LMX1A-U-R	5′-CAACCAAATCCAAACACACAAAAC-3′	MSP
LMX1A-BS06F	5′-AAGTTGATTTGAAATGTATTTAAGAGTGAG-3′	BS
LMX1A-BS06R	5′-TAAATATATAAAAAATAAATACCCTCCCTC-3′	BS
LMX1A -QMSPF	5′-TGGGACGCGGGATTGTAAATTTTAT-3′	Q-MSP
LMX1A -QMSPR	5′-AAACCCTCGAAACGTCTCTACAAAA-3′	Q-MSP
LMX1A-387F	5′-*TCAGAAGGGTGATGAGTTTGTCC*-3′	qRT-PCR
LMX1A-585R	5′-GGGGCGCTTATGGTCCTTG-3′	qRT-PCR
CDH1-1129F	5′-CCCACCACGTACAAGGGTC-3′	qRT-PCR
CDH1-1304R	5′-ATGCCATCGTTGTTCACTGGA-3′	qRT-PCR
CDH2-890F	5′-CAGATAGCCCGGTTTCATTTGA-3′	qRT-PCR
CDH2-1053R	5′-CAGGCTTTGATCCCTCAGGAA-3′	qRT-PCR
FN1-242F	5′- GAAGCCGAGGTTTTAACTGC -3′	qRT-PCR
FN1-327R	5′- ACCCACTCGGTAAGTGTTCC -3′	qRT-PCR
ID2-431F	5′- CCACTATTGTCAGCCTGCATC-3′	qRT-PCR
ID2-610R	5′- AGAAATCATGAACACCGCTTA-3′	qRT-PCR
GAPDH-943F	5′-ACCCACTCCTCCACCTTTGACG-3′	qRT-PCR
GAPDH-1107R	5′- TCTCTTCCTCTTGTGCTCTTG -3′	qRT-PCR
MYC-1201F	5′-GGCAAAAGGTCAGAGTCTGG-3′	qRT-PCR
MYC-1409R	5′-GTGCATTTTCGGTTGTTGC-3′	qRT-PCR

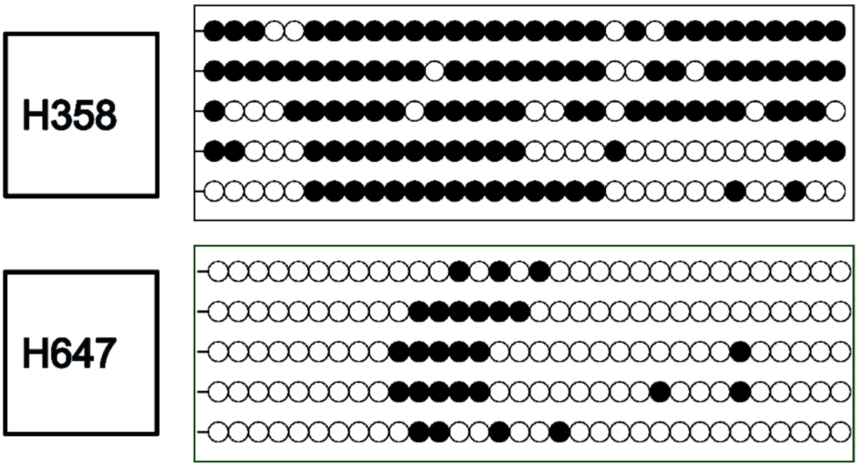

Figure S1. Bisulfite genomic sequencing analysis of the CpG sites located at the promoter region of *LMX1A*. The *LMX1A* methylation status in H358 and H647 cell lines was analyzed by bisulfite genomic sequencing. Each clone is represented by a row, and 32 CpG sites are represented as circles. Black circles and white circles represent methylated and unmethylated CpG sites, respectively.

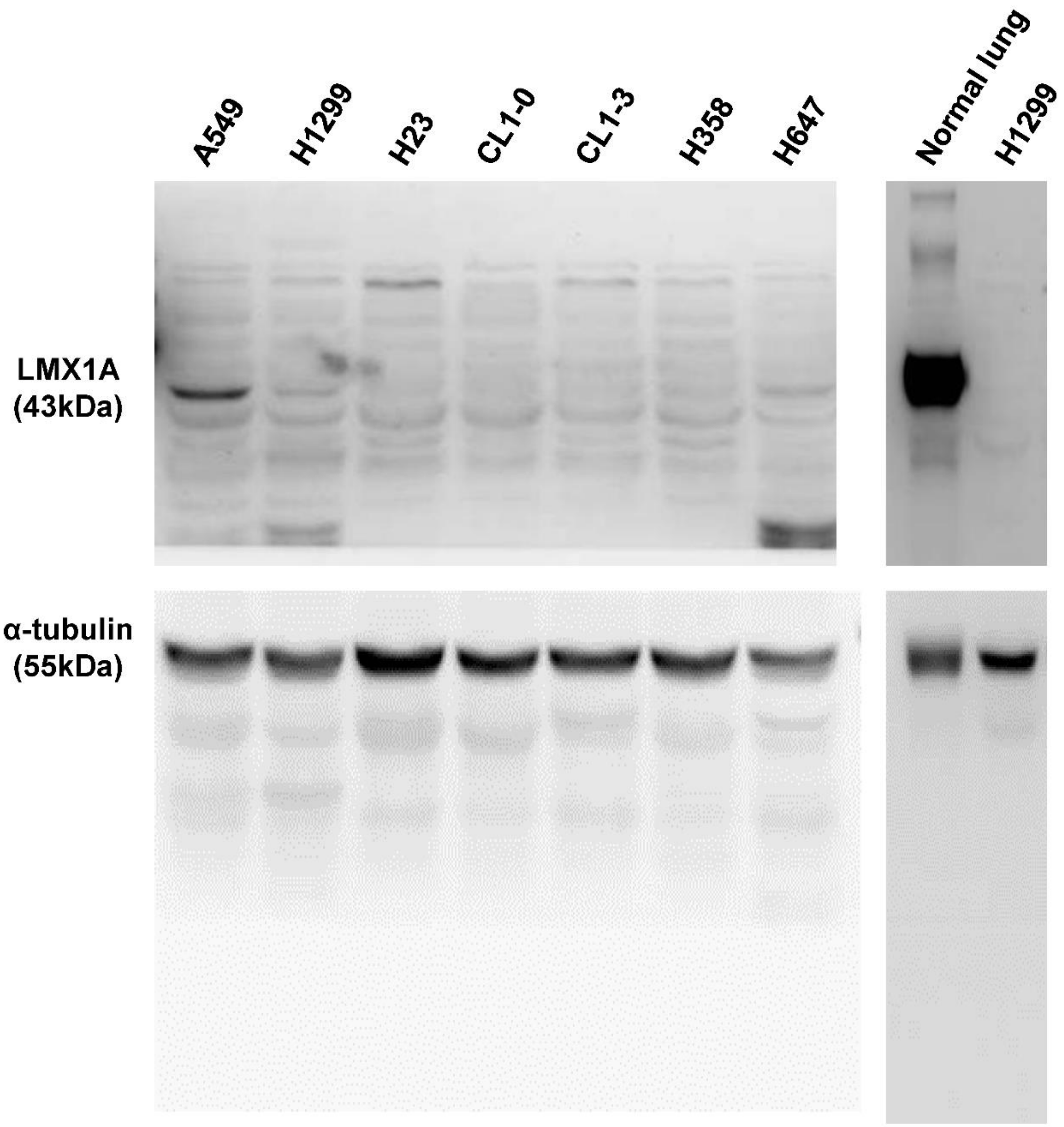

Figure S2-1. Protein expression of LMX1A in human NSCLC cells, normal lung tissues, and NSCLC cells with expression of LMX1A. LMX1A expression was analyzed by Western blotting in human NSCLC cell lines and normal lung tissues using an anti-LMX1A antibody. α-Tubulin was used as an internal control.

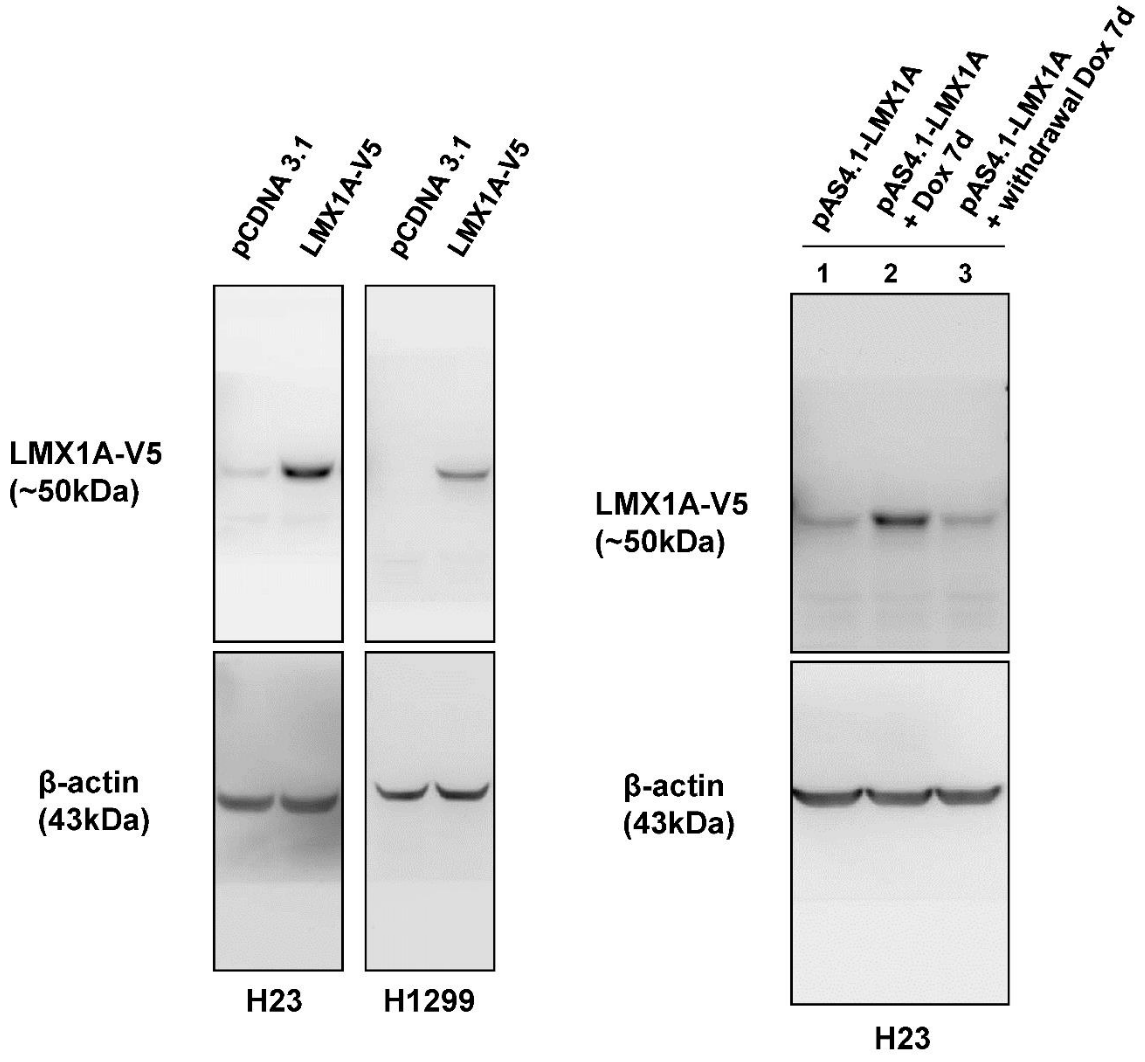

Figure S2-2. Protein expression of LMX1A in human NSCLC cells, normal lung tissues, and NSCLC cells with expression of LMX1A-V5. LMX1A expression was analyzed by Western blotting in human NSCLC cell lines and normal lung tissues using an anti-V5 antibody. β-Actin was used as an internal control.

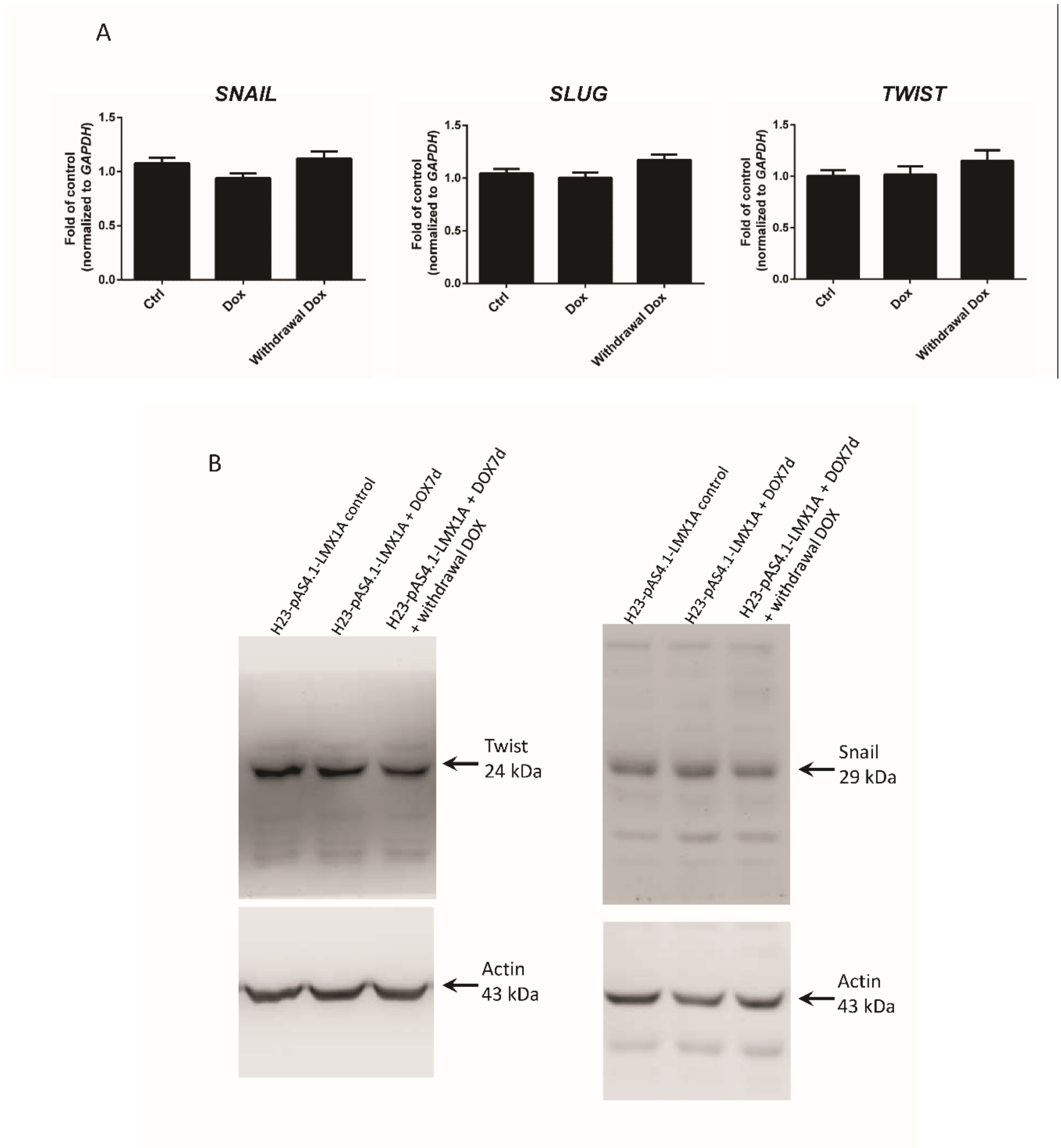

Figure S3. Effects of LMX1A on EMT-related transcription factors in NSCLC cells. The expression of EMT-related transcription factors (*SNAIL*, *SLUG*, and *TWIST*) was detected by qRT-PCR (A) and Western blotting (B) after induction of *LMX1A* in H23 cells. Data are shown as fold changes of mRNA expression relative to control cells without doxycycline. qRT-PCR are expressed as the mean ± SEM from three independent experiments, each conducted in duplicate. β-Actin was used as an internal control.

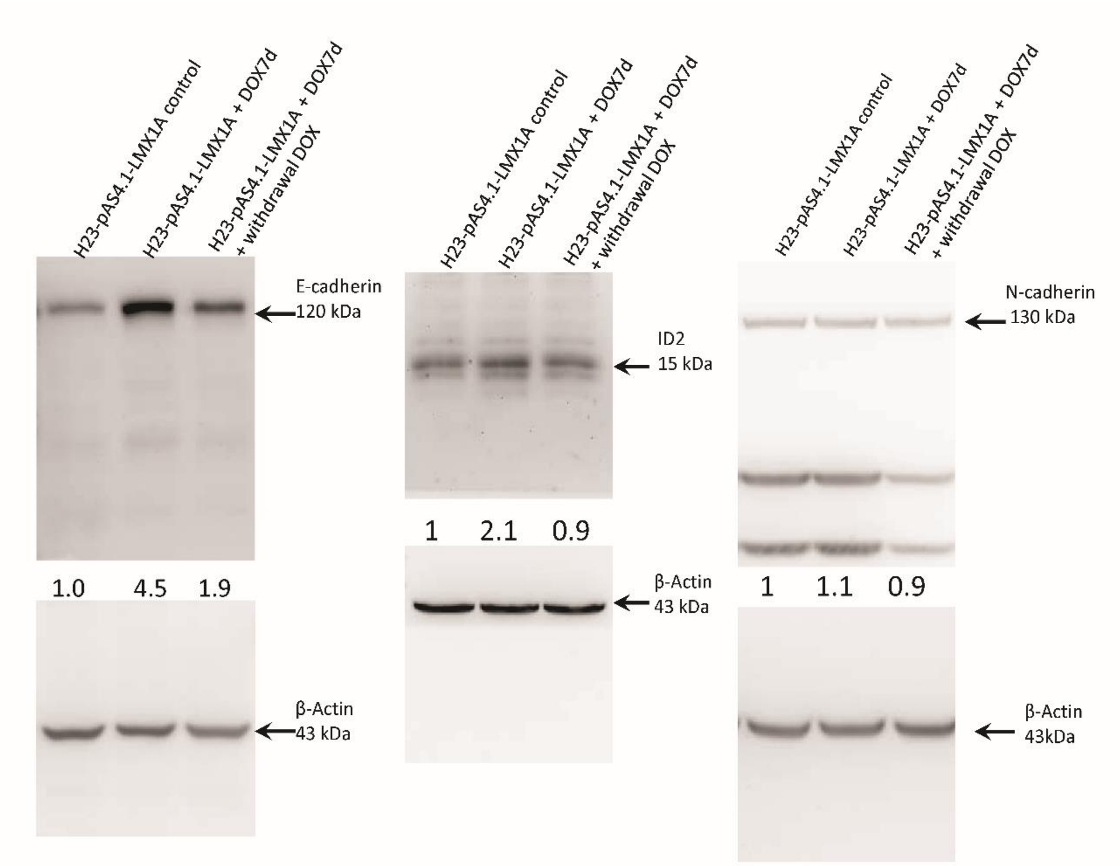

Figure S4. Effects of LMX1A on EMT-related genes in NSCLC cells. The protein expression of EMT-related genes (E-cadherin, ID2, and N-cadherin) was detected by Western blotting after induction of *LMX1A* in H23 cells. The numbers in the blot represent the ratios between and the EMT-related genes (E-cadherin, ID2, and N-cadherin) and internal control.

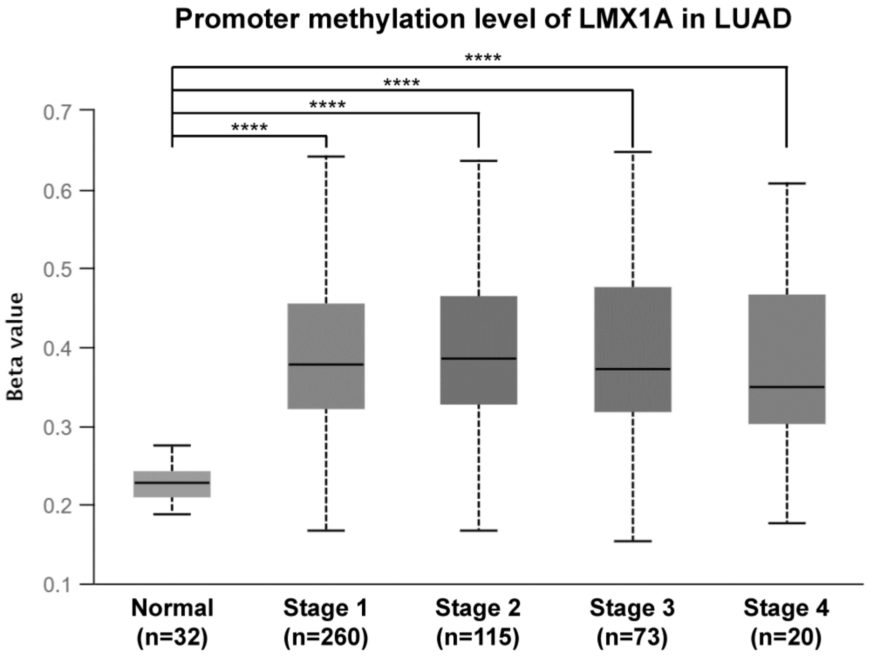

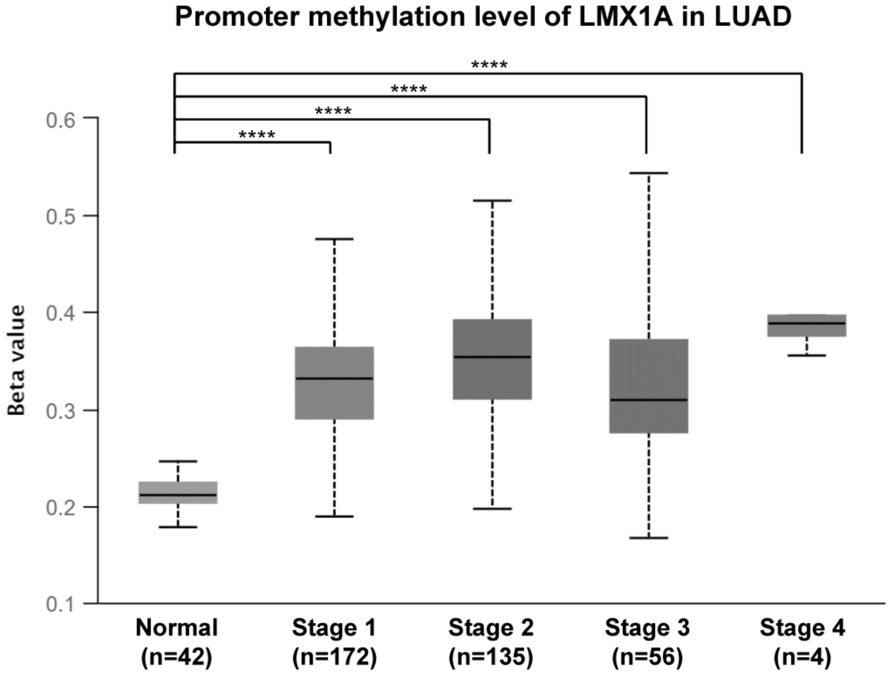

Figure S5. The methylation of *LMX1A* in NSCLC patients according to tumor stages, analyzed from The Cancer Genome Atlas (TCGA) database through the UALCAN (http://ualcan.path.uab.edu) web portal. The methylation of LMX1A in NSCLC patients occurred early in stage I patients and then maintained through all the stages. LUAD: Lung adenocarcinoma; LUSC: Lung squamous cell carcinoma. **** $p < 0.0001$ (Student's t-test).

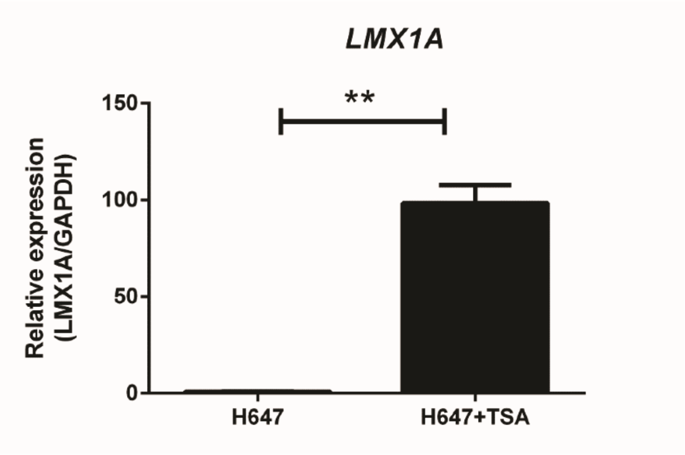

Figure S6. Reexpression of LMX1A after treatment of histone deacetylase (HDAC) inhibitors in H647 lung cancer cells. Cells were treated with the HDAC inhibitor (trichostatin A, TSA) for 1 day to restore LMX1A expression. The cells were then depleted of the drugs and cultured for 2 more days. qRT-PCRs are expressed as the mean ± SEM from two independent experiments, each conducted in duplicate. ∗∗ p < 0.01 (Mann–Whitney U test or Student's *t*-test).

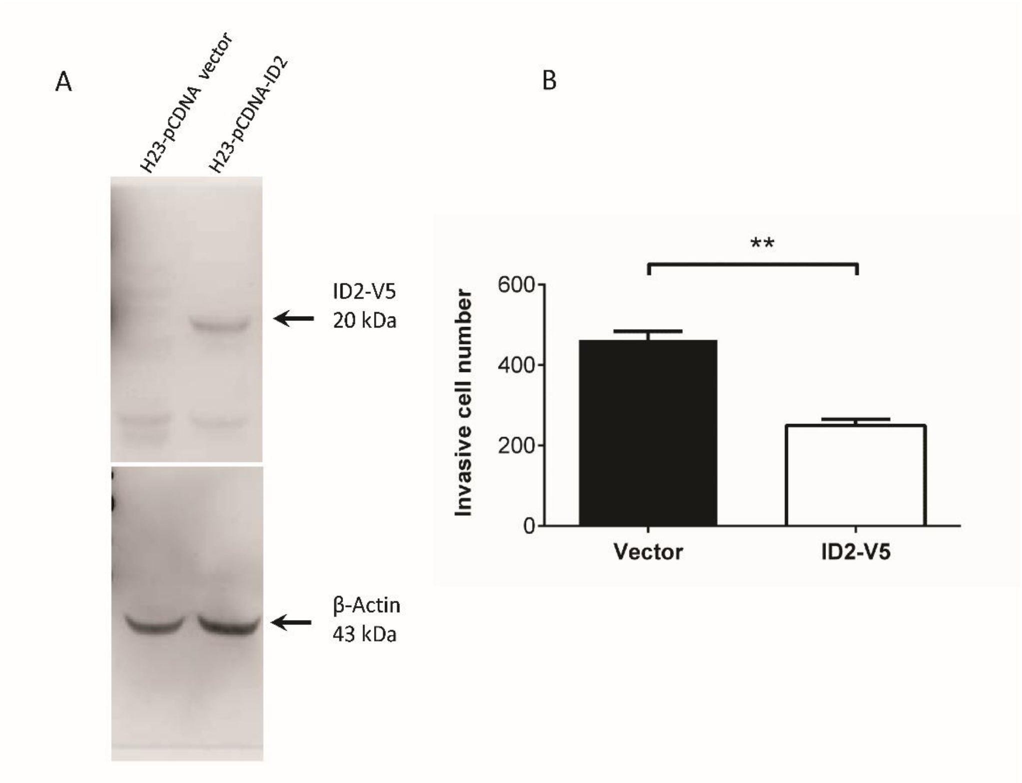

Figure S7. Transient overexpression of ID2 suppresses the invasion of cancer cells. (A) Expression of ID2 in H23 NSCLC cells transiently transfected with *ID2*-V5 or the empty vector (pCDNA3.1) was analyzed by Western blots using an anti-V5 antibody. β-Actin was used as an internal control. Matrigel invasion assays were performed using H23 cells expressing *ID2*-V5 or the empty vector. These results are presented as the mean ± SEM from two independent experiments in triplicate. ** $p <$ 0.01 (Mann–Whitney U test).

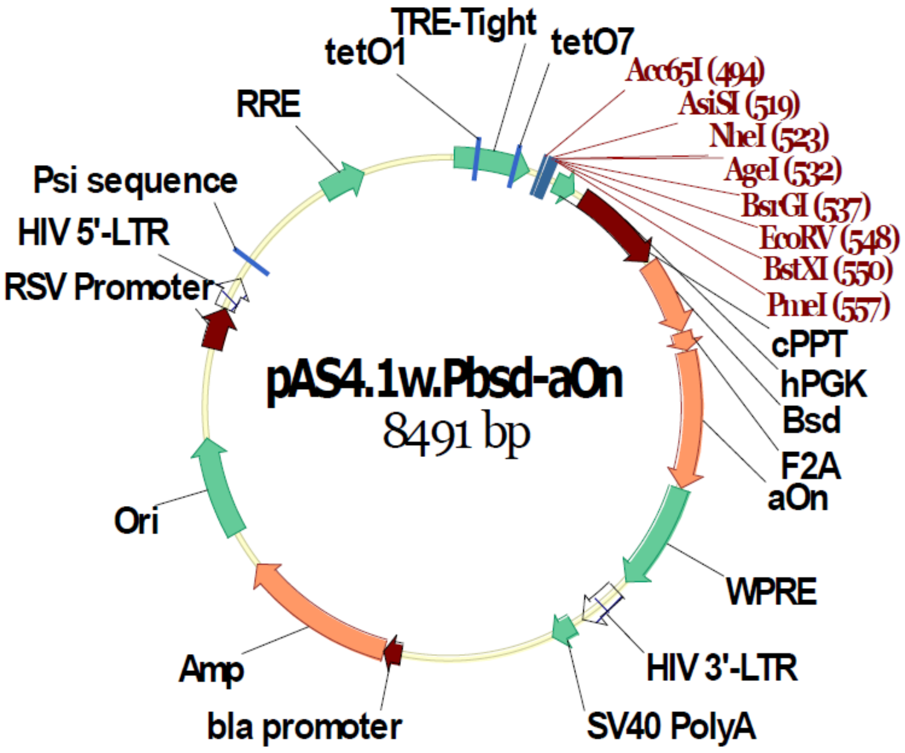

Figure S8. Map of pAS4.1w.Pbsd-aOn. pAS4.1w.Pbsd-aOn is an all-in-one tetracycline-inducible plasmid (TRE tight version that contains 7 copies of the modified tetO sequence, a tetracycline repressor binding sequence). ORF of *aOn* TRE transactivator (function as a transcriptional factor) was introduced into the downstream of the *bsd* gene by fusing it with the F2A sequence to link *bsd*ORF. The *aOn*ORF has the same reading frame as *bsd*; as a result, this fusion protein will be post-translationally cleaved by F2A protease into aOn and bsd functional proteins.

Supplementary Methods

Cell lines and protein extract of normal lung tissues

Eight human lung cancer cell lines (A549, H1437, H23, CL1-0, CL1-3, H358, H1299, and H647) were used in this study. They were a kind gift from Professor Yi-Ching Wang (National Cheng Kung University, Taiwan). Cells were cultured in Roswell Park Memorial Institute (*RPMI*) 1640 medium (GIBCO, Gaithersburg, MD, USA) supplemented with 2 mM L-glutamine, 1% nonessential amino acids (NEAA), 10% heat-inactivated fetal bovine serum (FBS), 100 U/ml penicillin, and 100 µg/ml streptomycin (GE Healthcare Life Sciences, Chicago, IL, United States). All cell cultures were grown as monolayer cultures and maintained in a humidified atmosphere containing 5% CO_2 in air at 37 °C. The protein extract of normal human lung tissues was commercially obtained from the biotechnology company (GenDiscovery, Taipei, Taiwan)

Western Blot Analysis

Protein was extracted from cells using ice-cold RIPA buffer (50 mM Tris HCl (pH 7.4), 150 mM NaCl, 1 mM EDTA, 1% (v/v) Triton-X 100, 0.1% (w/v) SDS) supplemented with phenylmethylsulfonyl fluoride (PMSF) and protease inhibitor cocktail (2 mM AEBSF, 1 mM EDTA, 130 µM bestatin, 14 µM E-64, 1 µM leupeptin, 0.3 µM aprotinin). Protein concentrations were determined using a bicinchoninic acid (BCA) assay according to the manufacturer's instructions. Equivalent amounts of protein were separated on 12% SDS-PAGE gels and transferred to polyvinylidene difluoride (PVDF) membranes (Millipore, Billerica, MA, USA). Membranes were blocked at room temperature with 5% nonfat milk in Tris-buffered saline (TBS) containing 0.1% Tween 20 (TBS-T) for 1 h. Primary antibodies were diluted 1:2000 and incubated overnight at 4 °C. Secondary antibodies diluted 1:5000

were added and incubated at room temperature for 1 h. Signals were detected using ECL detection reagent (Millipore) following the manufacturer's instructions. The primary antibodies used were as follows: Mouse anti-V5 (Bio-Rad Laboratories, Taiwan), anti-LMX1A (GTX77819, GeneTex, CA, USA), anti-TWIST (ab49254; abcam, Cambridge, MA, USA), anti-SNAIL (GTX100754; GeneTex, Irvine, CA, USA), anti-E-cadherin (610405; BD Biosciences, San Jose, CA, USA), anti-N-cadherin (sc-6260; Santa Cruz Biotechnology, Santa Cruz, CA, USA), anti-ID2 (AF4660, R&D, Minneapolis, MN , USA), and rabbit anti-β-actin (GeneTex, CA, USA). Horseradish peroxidase-conjugated rabbit anti-mouse or goat anti-rabbit secondary antibodies (GeneTex, CA, USA) were used as appropriate.

MTS Prolifeation Assay

MTS assays were performed with the Cell Titer 96 AQ One Solution Cell Proliferation Assay (Promega Corporation, Madison, WI) (17). MTS reagent (20 µl/well) was added to 100 µL of medium containing cells in each well of 96-well plates and left for 4 h at 37 °C under humidified 5% CO_2 in air. For colorimetric analysis, the absorbance at 490 nm was recorded using a microplate reader. Each condition was repeated at least three times. Total cells were harvested at the designated times after treatment.

Colony formation

Cells were trypsinized and resuspended in 1.5 ml of 0.5% agarose, and then poured onto a 1.5 ml 1% agarose bed in 35 mm tissue culture dishes. After 3–4 weeks, cells were stained with a solution containing 0.005% crystal violet, 1.9% formaldehyde, and 0.15 M NaCl, for 30 min. After washing and drying, colonies larger than 1 mm were counted.

Invasion assay

In vitro cell invasion was determined in 24-well Transwell plates (Coster) with a Matrigel (BD Biosciences) coating. Stable transfectants expressing *LMX1A* were suspended in optimum medium at a concentration of 1~5 x 10^4 cells/ml, placed in the upper chambers, and incubated for 16–24 h at 37 °C in 5% CO_2. Noninvasive cells in the upper chamber were subsequently removed with a cotton-tip applicator. Invasive cells on the lower surface were fixed with methanol and stained with hematoxylin. The number of invasive cells were determined by counting 10 high-power fields (100× magnification) on each membrane and calculated as the mean number of cells per field. All cell lines were assayed in duplicate for each experiment, and each experiment was repeated three times.

Transfection

Cells plated in 100 mm dishes were transfected at 50–80% confluence with LMX1A expression vectors or with control vectors, using the liposome-mediated transfection method. To establish cells stably expressing LMX1A, H23 and H1299 cells were transfected with the plasmid of choice (pcDNA3.1-LMX1A or pAS4.1-LMX1A) for two days, and then trypsinized and plated at low density. Stable mixed clones were selected by maintaining cells in medium containing the G418 or blasticidin antibiotic.
